# Prevalence Patterns and Onset Prediction of High Myopia for Children and Adolescents in Southern China via Real-World Screening Data: Retrospective School-Based Study

**DOI:** 10.2196/39507

**Published:** 2023-03-01

**Authors:** Jieying Guan, Yingting Zhu, Qiuyue Hu, Shuyue Ma, Jingfeng Mu, Zhidong Li, Dong Fang, Xiaohua Zhuo, Haifei Guan, Qianhui Sun, Lin An, Shaochong Zhang, Peiwu Qin, Yehong Zhuo

**Affiliations:** 1 State Key Laboratory of Ophthalmology, Zhongshan Ophthalmic Center, Sun Yat-sen University, Guangdong Provincial Key Laboratory of Ophthalmology and Visual Science Guangdong Provincial Clinical Research Center for Ocular Diseases Guangzhou China; 2 Institute of Biopharmaceutical and Health Engineering, Shenzhen International Graduate School, Tsinghua University Shenzhen China; 3 Shenzhen Eye Hospital, Jinan University, Shenzhen Eye Institute Shenzhen China

**Keywords:** vision screening, high myopia, prevalence, random forest, children, adolescents

## Abstract

**Background:**

Patients with high myopia have an increased lifetime risk of complications. The prevalence patterns of high myopia in children and adolescents in southern China are unclear. Early identification of high-risk individuals is critical for reducing the occurrence and development of high myopia and avoiding the resulting complications.

**Objective:**

This study aimed to determine the prevalence of high myopia in children and adolescents in southern China via real-world screening data and to predict its onset by studying the risk factors for high myopia based on machine learning.

**Methods:**

This retrospective school-based study was conducted in 13 cities with different gross domestic products in southern China. Through data acquisition and filtering, we analyzed the prevalence of high myopia and its association with age, school stage, gross domestic product, and risk factors. A random forest algorithm was used to predict high myopia among schoolchildren and then assessed in an independent hold-out group.

**Results:**

There were 1,285,609 participants (mean age 11.80, SD 3.07, range 6-20 years), of whom 658,516 (51.2%) were male. The overall prevalence of high myopia was 4.48% (2019), 4.88% (2020), and 3.17% (2021), with an increasing trend from the age of 11 to 17 years. The rates of high myopia increased from elementary schools to high schools but decreased at all school stages from 2019 to 2021. The coastal and southern cities had a higher proportion of high myopia, with an overall prevalence between 2.60% and 5.83%. Age, uncorrected distance visual acuity, and spherical equivalents were predictive factors for high myopia onset in schoolchildren. The random forest algorithm achieved a high accuracy of 0.948. The area under the receiver operator characteristic curve (AUC) was 0.975. Both indicated sufficient model efficacy. The performance of the model was validated in an external test with high accuracy (0.971) and a high AUC (0.957).

**Conclusions:**

High myopia had a high incidence in Guangdong Province. Its onset in children and adolescents was well predicted with the random forest algorithm. Efficient use of real-world data can contribute to the prevention and early diagnosis of high myopia.

## Introduction

Patients with high myopia have an increased lifetime risk of complications such as cataract formation, retinal detachment from retinal holes or tears, myopic foveoschisis, peripapillary deformation, dome-shaped macula, myopic choroidal neovascularization, and glaucoma [1]. The number of patients with high myopia is increasing annually and will reach 938 million people by 2050 [2], which could lead to significant global productivity loss, with East Asia having the highest potential burden [3].

In the clinic, parents usually ask questions such as “will my child develop high myopia in the coming year” and “what is the possibility of the myopia of my child developing into high myopia in the future” even though their child has undergone only one ophthalmic examination. While it is well known that the risk of high myopia is relatively high in children with myopia onset during early school age [4], predicting the specific possibility of myopia development in a certain child is difficult for ophthalmologists.

The answer could be obtained by evaluating real-world screening of large-scale data. China promulgated guidelines for myopia prevention and control in children and adolescents in 2021 [5], emphasizing the role of regular vision screening and electronic vision health records to improve the prediction, prevention, early control, and the diagnostic accuracy of myopia by screening for visual acuity (VA) and noncycloplegic autorefraction (NCR) in schools. The determination of VA combined with NCR is a clinically appropriate and low-cost methodology that may provide a model for screening and for epidemiological studies [6,7].

Artificial intelligence (AI) may help in obtaining useful information from this screening data. Wang et al [8] trained and validated a random forest algorithm to yield uncorrected distance visual acuity (UDVA) and spherical equivalents (SE) that predict myopia progression in children. Lin et al [9] developed an algorithm to predict SE and the onset of high myopia up to 8 years ahead by considering variables including age, SE, and annual progression rate. Furthermore, some researchers detected myopia using machine learning based on ocular images [10-12]. However, these studies have not attempted to retrospectively predict the risk of high myopia using large-scale screening data. 

Our study describes the prevalence patterns of high myopia from vision-screening data obtained from millions of children and adolescents in southern China. It also illustrates the method for using real-world screening data in research. An AI model aided in identifying the risk factors for high myopia occurrence and predicting the onset of high myopia at an early stage with high accuracy using real-world data for the first time.

## Methods

### Ethics Statement

This retrospective and school-based study was approved by the institutional review board or ethics committee of Sun Yat-sen University, Guangzhou, China (2021KYPJ185), and was registered with the Chinese Clinical Trial Registry (ChiCTR; identifier: ChiCTR2200057391). The need for consent was waived because all data sets were deidentified before transfer for data analysis.

### Study Population and Data Source

Myopia screening was conducted in Guangdong Province, southern China, which included 21 cities with 115.21 million permanent residents. Guangdong’s gross domestic product (GDP) increased to 12,436.967 billion in 2021 [13]. Using electronic vision screening from Guangdong Eyevision Medical Technology Co, Ltd, the data collected included student ID, name, sex, location, class, grade, school type, UDVA, NCR, systolic pressure, diastolic pressure, height, weight, sexual maturity, and other ophthalmic diseases or surgeries. Sexual maturity was defined as the occurrence of menstruation or spermatorrhea.

### Screening Procedures

The staff of the participating schools received training from the administration, and local experienced certified optometrists performed the ocular examinations. UDVA was assessed monocularly using a Chinese standard logarithm VA chart (GB11533-2011) in an illuminated cabinet (WSVC-100, Wenzhou, China). LogMAR VA scores were recorded. Eyesight was measured at least thrice without cycloplegia using Topcon KR-1 (Topcon Co, Tokyo, Japan). Measurements were repeated if one measurement deviated from the other two by >0.5 D. Three reliable measurements were averaged for analysis.

### Inclusion Criteria and Outcome Definitions

Our screening data encompassed 13 cities in Guangdong Province (Guangzhou, Shenzhen, Foshan, Zhuhai, Huizhou, Qingyuan, Heyuan, Shanwei, Shantou, Shaoguan, Yangjiang, Yunfu, and Jieyang). The cities have different populations, GDPs, and locations that show broad statistical variation as representing the whole province. Children and adolescents aged 6-20 years were recruited between 2019 and 2021. The right eye was arbitrarily selected to represent a specific individual. Individuals with missing records, other eye diseases, or eye surgeries were excluded.

Noncycloplegic SE was calculated in accordance with the standard formula: SE = sphere + ½ × cylinder. Screening myopia was defined as a UDVA of ≥0 logMAR and an SE of ≤–0.5 D. It was further subdivided into low myopia (–6 D<SE≤–0.5 D) and high myopia (SE≤–6 D) according to the IMI myopia definition and classification [14]. In the cohort study, high myopia progression was considered a shift in SE from the low myopia range toward the high myopia window during observation. BMI was calculated as the weight (in kg) divided by the square of the height (in m). Mean arterial pressure (MAP) was calculated as follows: MAP = diastolic pressure + 1/3 × (systolic pressure – diastolic pressure). The 13 cities were divided into 3 levels based on GDP: first-level cities with a per capita GDP of >100,000, second-level cities with a GDP between 50,000 and 100,000, and third-level cities with a GDP of <50,000.

### Data Processing and Feature Selection

**Figure 1:**
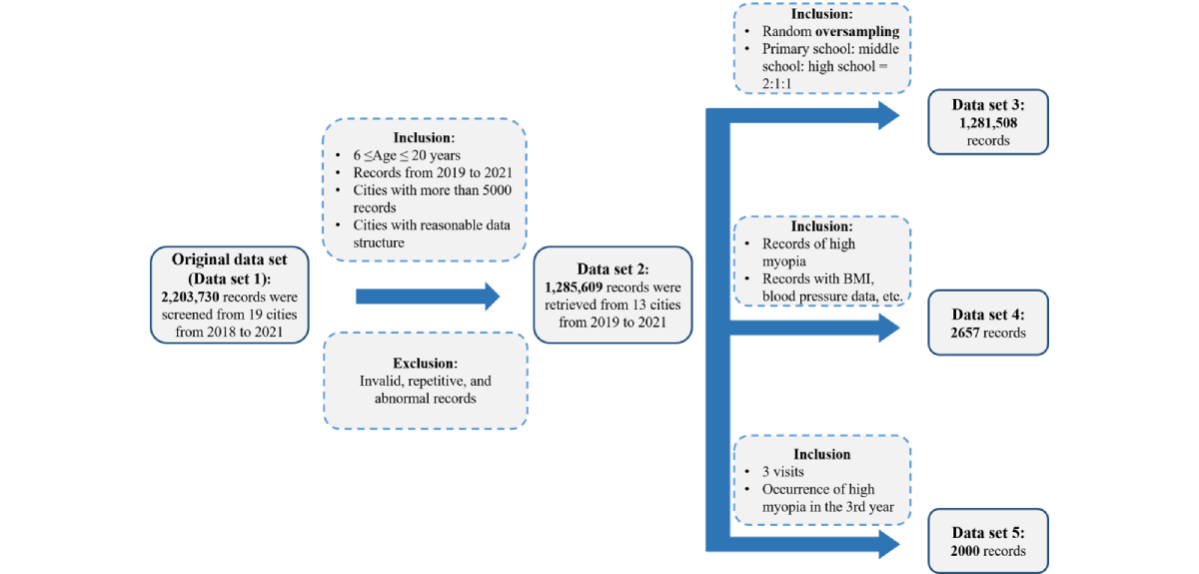
Flowchart for quality control for the data set.

Figure 1 shows a flowchart for quality control for the data sets in this study. The first step was to compile an original data set from the electronic vision-screening data (data set 1). The next step was cleaning data set 1 and manually removing data that were not qualified for analysis because of invalidation, repetition, and abnormality. Only 1,285,609 records from 13 cities between 2019 and 2021 that met the inclusion criteria were included in data set 2. Data set 2 was used to describe the prevalence patterns of high myopia among different age groups, sexes, school stages, and GDPs.

To visualize the relationship between high myopia and per capita GDP in cities in Guangdong Province from 2019 to 2021, we applied the random oversampling method to balance the distribution in data set 2. By selecting the ratio of primary school to middle school to high school as 2:1:1 in each city, we successfully obtained a newly balanced data set 3.

Additionally, 2657 participants with high myopia and complete data were selected from data set 2 to determine risk factors and form data set 4. The SE value of the right eye was used in multivariable linear regression analysis. Six factors, namely age, sex, BMI, MAP, sexual maturity, and per capita GDP, included in the screening data, were selected as potential risk factors. Permutation importance was used to measure the influence of the factors on the predicted results.

In data set 2, a total of 16,760 participants with a 3-year examination period were selected, of whom 380 participants presented high myopia during observation and 14,948 did not present high myopia in the 3 years. Considering this imbalance between participants (approximate 1:39), we randomly selected 1000 records from 380 subjects with replacement (random oversampling) and 1000 records from 14,948 subjects without replacement (random undersampling) to reach a new balance. The main outcome was high myopia onset, scored as 0 or 1, with 0 representing negative samples and 1 representing positive samples. Finally, data set 5 was formed, which included 2000 records to predict the onset of high myopia.

### Algorithm Development and Evaluation Metrics

Data set 5 was randomly split into a training set and a test set at a 4:1 ratio; a 5-fold cross-validation (CV) approach was used during training. Several machine learning algorithms, including logistic regression, a support vector machine, a k-nearest neighbor algorithm, and a random forest algorithm were applied to derive the classification results. Accuracy, precision, and recall were calculated to assess the prediction results for the training, CV, and test sets, respectively. Precision represents the percentage of true positive predictions among all positive samples, whereas recall indicates the percentage of true positive predictions among all correct predictions. Since we aimed to raise an early warning for cohorts at risk, recall was more persuasive than precision. The receiver operator characteristic (ROC) curve described the relationship between the true and false positive rates by setting different probability values as classification thresholds. A larger area under the ROC curve (AUC) indicated better efficacy. The Kolmogorov-Smirnov (K-S) curve assessed the model’s performance. The largest distance between the 2 curves (K-S value) was calculated for the train, CV, and test sets separately. Finally, the model’s performance was validated by applying it to an independent hold-out group from Shenzhen Eye Hospital. These records, available for external validation, were obtained from 2547 individuals with a 3-year follow-up from 2019 to 2021 (age range 6-16, mean 8.78, SD 2.39 years), of whom 47 subjects exhibited high myopia and 2500 did not during the 3 years of observation.

### Statistical Analysis

Data were processed and analyzed using the R programming language [15] and Python 3.8 [16]. Continuous variables are shown as mean (SD) or median (IQR) and categorical variables as frequencies. The normal distribution was tested using visual response and K-S and Shapiro-Wilk tests. Comparisons were performed using independent samples *t* tests, 1-way ANOVA, the Kruskal-Wallis test, or the chi-square test, as appropriate. All *P* values were 2-tailed, where *P*<.05 was statistically significant.

## Results

### Characteristics of the Study Population

In total, 1,285,609 participants were obtained in the 3 years of screening (Table 1). There were 658,516 (51.2%) males. The average age was 11.80 (SD 3.07, range 6-20) years. The incidence of high myopia differed in 2019 and 2021, with females having a higher rate (for both years, *P*<.001), but not in 2020 (*P*=.70). The overall prevalence of high myopia was 4.48% (2019), 4.88% (2020), and 3.17% (2021).

**Figure 2:**
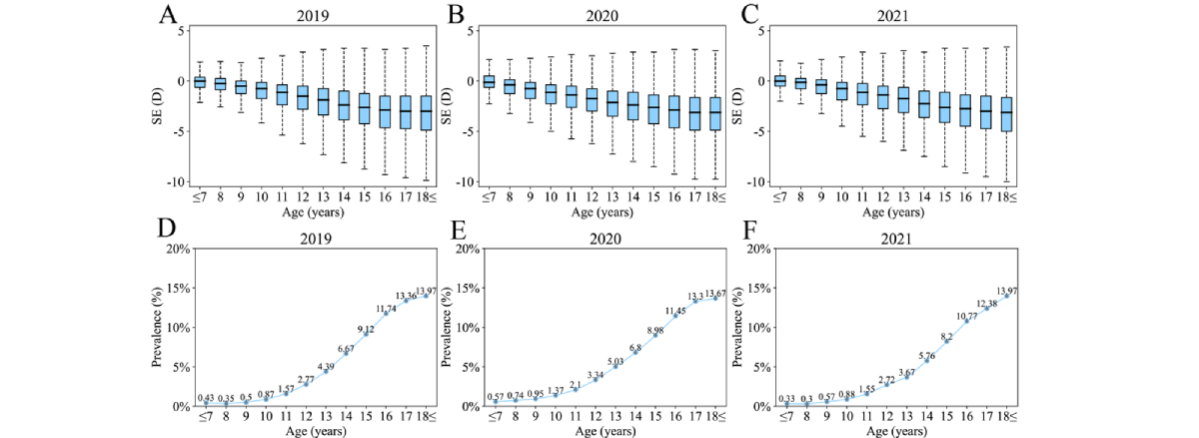
Spherical equivalent distribution and high myopia prevalence among children and adolescents. (A)-(C) Spherical equivalent distribution for school students aged 6 to 20 years, respectively, from 2019 to 2021. (D)-(F) High myopia prevalence for school students aged 6 to 20 years, respectively, from 2019 to 2021. D: diopter; SE: spherical equivalent.

**Figure 3:**
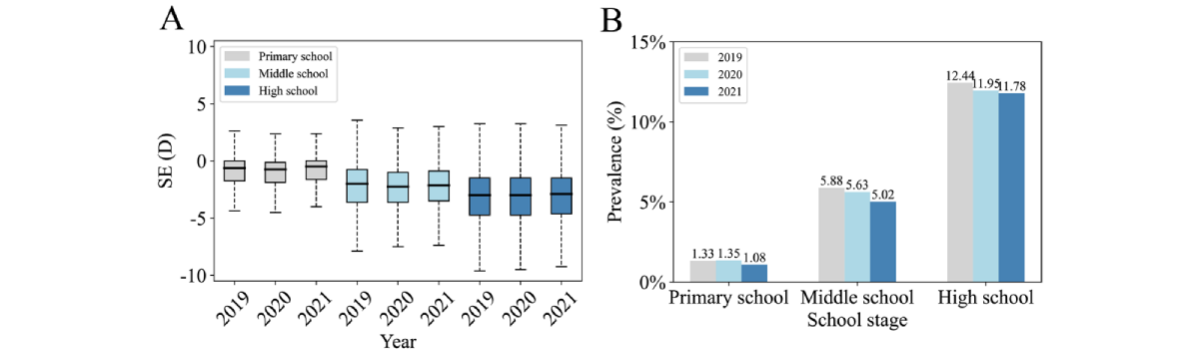
Spherical equivalent distribution and high myopia prevalence for different school stages. (A) Spherical equivalent distribution for different school stages, respectively, from 2019 to 2021. (B) High myopia prevalence for different school stages, respectively, from 2019 to 2021. D: diopter; SE: spherical equivalent.

Figure 2 shows that the rate of high myopia increased exponentially with age, from 0.43%, 0.57%, and 0.33% under 7 years to 13.97%, 13.67%, and 13.97% over 18 years in 2019, 2020, and 2021, respectively. There was an increasing trend in the prevalence of high myopia from the age of 11 to 17 years, with an annual growth of >1%. The 3-year overall prevalence of high myopia was 1.25% (95% CI 1.23%-1.28%), 5.60% (95% CI 5.53%-5.68%), and 12.09% (95% CI 11.95%-12.22%) among participants in primary, middle, and high schools, respectively (*P*<.001). However, the rate of high myopia declined annually. Specifically, the prevalence over this period decreased from 1.33% to 1.08%, 5.88% to 5.02%, and 12.22% to 11.78% among participants in primary, middle, and high schools, respectively (for all, *P*<.001, Figure 3).

There were significant differences in the prevalence of high myopia in cities with different per-capita GDP levels. The high myopia rate in cities with the second-GDP-level cities was significantly higher than that in the first- and third-level cities for all 3 years (*P*<.001); in first-level cities, it declined from 4.28% in 2019 to 1.12% in 2021 (*P*<.001). However, high myopia in third-level cities increased from 2.46% (2019) to 3.33% (2021; *P*<.001) but was stable in second-level cities between 5.03% and 5.38%.

**Table 1:** Demographic characteristics of the study participants with high myopia (N=1,285,609).

Variables			2019							2020							2021				
			Count, n		Spherical equivalent (D), mean (SD)		High myopia (%), measure (95% CI)		Count, n			Spherical equivalent (D), mean (SD)		High myopia (%), measure (95% CI)		Count, n			Spherical equivalent (D), mean (SD)		High myopia (%), measure (95% CI)
**Sex**																					
	Male	235,007		–1.67 (2.08)		4.29 (4.21-4.37)		241,506			–1.93 (2.13)		4.89 (4.80-4.98)		182,003			–1.38 (1.96)		3.04 (2.96-3.12)	
	Female	209,569		–1.87 (2.09)		4.70 (4.61-4.79)		248,374			–2.03 (2.10)		4.87 (4.78-4.95)		169,150			–1.57 (1.97)		3.31 (3.22-3.39)	
**Age (years)**																					
	≤7^a^	38,386		–0.17 (1.22)		0.43 (0.36-0.50)		41,712			–0.16 (1.38)		0.57 (0.49-0.64)		33,434			–0.11 (1.18)		0.33 (0.27-0.40)	
	8	40,688		–0.41 (1.24)		0.35 (0.30-0.41)		35,078			–0.63 (1.53)		0.74 (0.65-0.82)		37,305			–0.34 (1.23)		0.30 (0.25-0.36)	
	9	41,642		–0.72 (1.38)		0.50 (0.43-0.57)		36,742			–1.01 (1.60)		0.95 (0.85-1.05)		45,932			–0.69 (1.38)		0.57 (0.51-0.64)	
	10	42,885		–1.08 (1.51)		0.87 (0.79-0.96)		42,897			–1.38 (1.66)		1.37 (1.26-1.48)		41,247			–1.08 (1.54)		0.88 (0.79-0.97)	
	11	45,887		–1.47 (1.66)		1.57 (1.45-1.68)		50,681			–1.71 (1.76)		2.10 (1.97-2.22)		42,386			–1.43 (1.69)		1.55 (1.43-1.67)	
	12	46,150		–1.84 (1.82)		2.77 (2.62-2.92)		56,483			–2.05 (1.85)		3.34 (3.19-3.49)		40,926			–1.77 (1.86)		2.72 (2.57-2.88)	
	13	42,664		–2.23 (1.95)		4.39 (4.20-4.59)		55,544			–2.37 (1.97)		5.03 (4.85-5.21)		31,113			–2.05 (1.90)		3.67 (3.46-3.88)	
	14	38,387		–2.58 (2.07)		6.67 (6.42-6.92)		51,829			–2.64 (2.06)		6.80 (6.58-7.02)		21,802			–2.44 (2.04)		5.76 (5.45-6.07)	
	15	34,206		–2.94 (2.17)		9.12 (8.81-9.42)		45,148			–2.94 (2.14)		8.98 (8.72-9.25)		19,501			–2.81 (2.14)		8.20 (7.81-8.58)	
	16	29,152		–3.16 (2.26)		11.74 (11.37-12.10)		38,162			–3.18 (2.23)		11.45 (11.13-11.77)		16,653			–3.05 (2.24)		10.77 (10.30-11.24)	
	17	25,796		–3.29 (2.32)		13.36 (12.94-13.77)		25,085			–3.37 (2.26)		13.30 (12.88-13.72)		12,774			–3.23 (2.28)		12.38 (11.81-12.96)	
	≥18^b^	18,733		–3.32 (2.36)		13.97 (13.47-14.47)		10,519			–3.36 (2.27)		13.67 (13.01-14.33)		8080			–3.38 (2.30)		13.97 (13.22-14.73)	
**School stage**																					
	Primary school	249,557		–1.04 (1.65)		1.33 (1.28-1.37)		232,225			–1.11 (1.73)		1.35 (1.31-1.40)		243,635			–0.93 (1.61)		1.08 (1.04-1.12)	
	Middle school	116,591		–2.35 (2.10)		5.88 (5.75-6.02)		158,703			–2.47 (2.01)		5.63 (5.51-5.74)		61,756			–2.34 (2.00)		5.02 (4.85-5.19)	
	High school	78,428		–3.21 (2.29)		12.22 (12.21-12.67)		98,952			–3.24 (2.22)		11.95 (11.75-12.15)		45,762			–3.18 (2.25)		11.78 (11.49-12.08)	
**City categories**																					
	GDP^c^≥10^d^	288,753		–1.69 (2.05)		4.28 (4.20-4.35)		19,273			–1.37 (2.02)		3.38 (3.12-3.63)		160,729			–0.92 (1.58)		1.12 (1.06-1.17)	
	10>GDP>5^e^	128,152		–2.08 (2.16)		5.38 (5.25-5.50)		437,604			–2.04 (2.11)		5.03 (4.96-5.09)		145,878			–2.13 (2.13)		5.38 (5.26-5.49)	
	GDP≤5^f^	27,671		–1.09 (1.91)		2.46 (2.28-2.64)		33,003			–1.51 (2.05)		3.78 (3.58-3.99)		44,546			–1.32 (2.01)		3.33 (3.17-3.50)	

^a^The minimum age was 6 years.

^b^The maximum age was 20 years.

^c^GDP: gross domestic product.

^d^Including 4 cities: Shenzhen, Zhuhai, Guangzhou, Foshan.

^e^Including 2 cities: Huizhou and Yangjiang.

^f^Including 7 cities: Heyuan, Jieyang, Qingyuan, Shantou, Shanwei, Shaoguan, and Yunfu.

### Prevalence of High Myopia Stratified by Per Capita GDP

**Figure 4:**
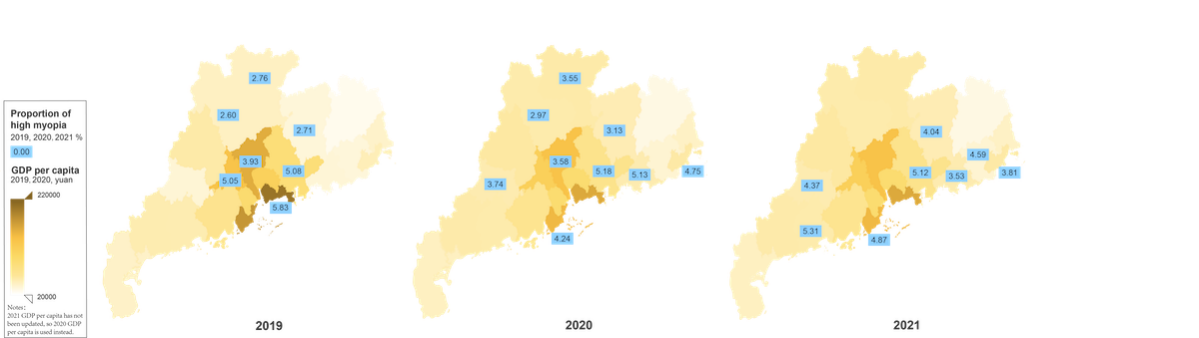
The proportion of cities with high myopia by GDP per capita level in Guangdong Province, China. GDP: gross domestic product.

**Figure 5:**
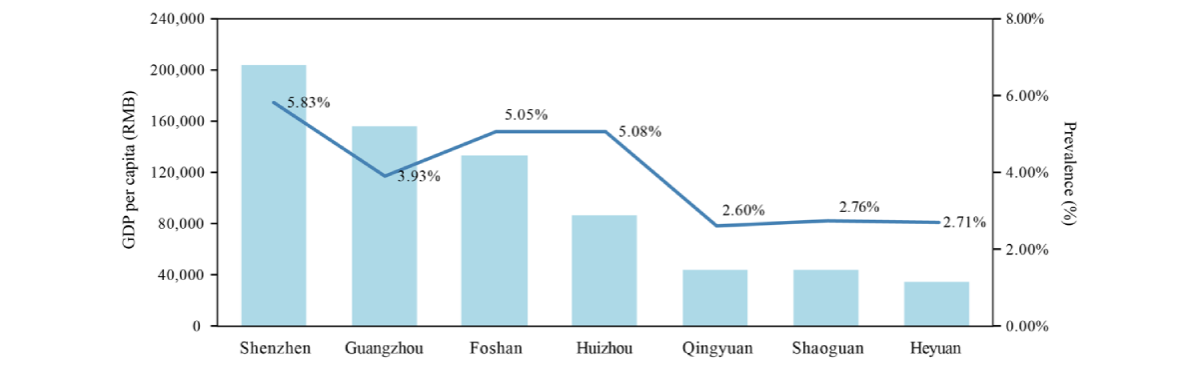
Prevalence of high myopia and the per capita GDP in the cities of Guangdong province, China, in 2019. GDP: gross domestic product; RMB: renminbi.

Figure 4 shows us the proportion of high myopia and economic development levels in southern China. The proportion of children and adolescents with high myopia varied between 2.60% and 5.83% in 2019, 2.97% and 5.18% in 2020, and 3.53% and 5.31% in 2021 with coastal and southern cities more affected. In 2019, before the COVID-19 pandemic, the rate of high myopia correlated with the economic level (*r*=0.83, *P*=.02), with Guangzhou (the capital city) having a rate of 3.93% (Figure 5). However, there was a weak correlation between 2020 and 2021 (*r*=0.05 and 0.41, respectively; for both *P*>.05).

### Determining the Risk Factors for High Myopia

**Figure 6:**
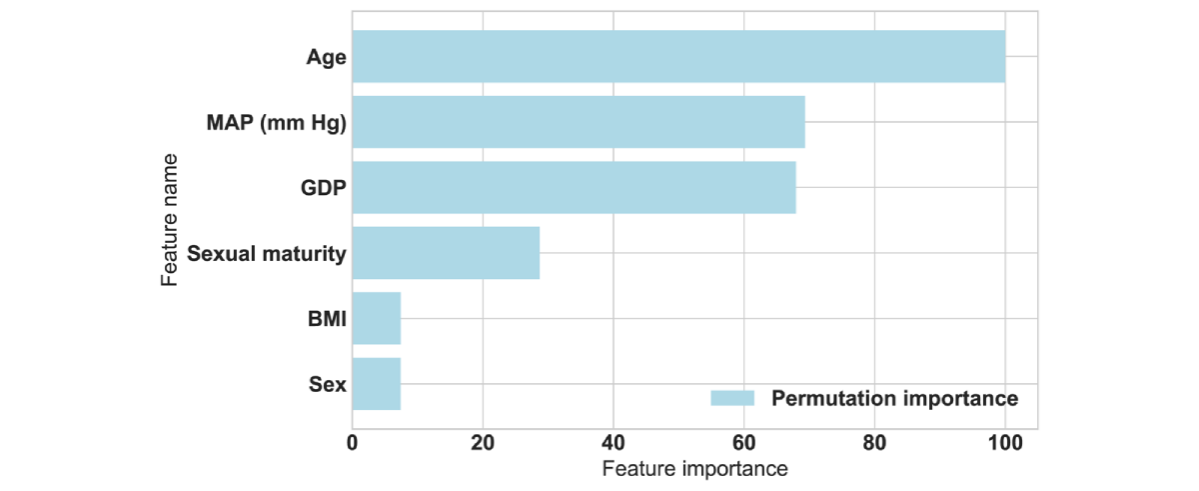
Important feature analysis. GDP: gross domestic product; MAP: mean arterial pressure.

Figure 6 shows that age was a significant risk factor for the progression of high myopia with the highest permutation importance, followed by MAP and GDP. Sexual maturity, BMI, and sex were not significantly correlated with high myopia.

### Prediction for the Onset of High Myopia

**Figure 7:**
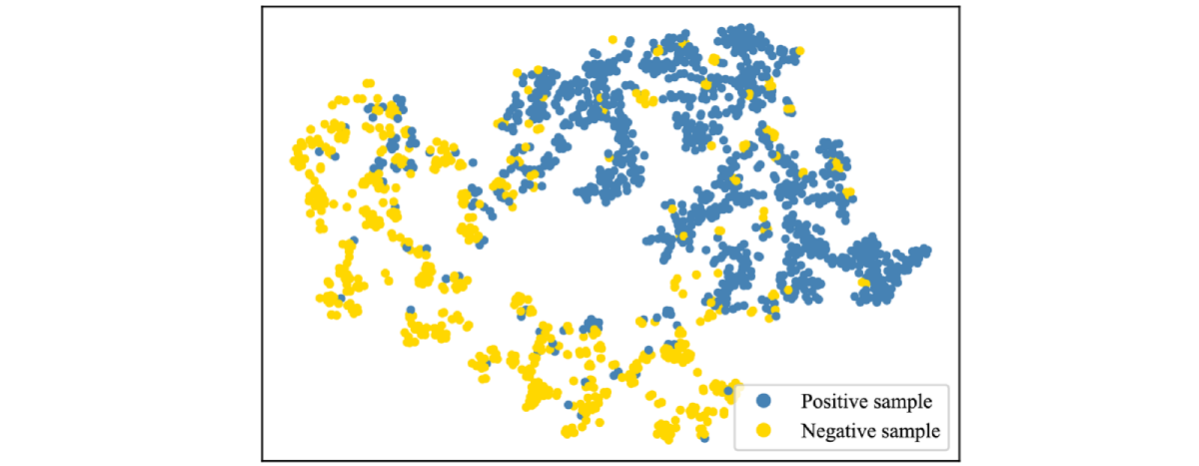
Visualization of standardized data by t-distributed stochastic neighbor embedding.

**Figure 8:**
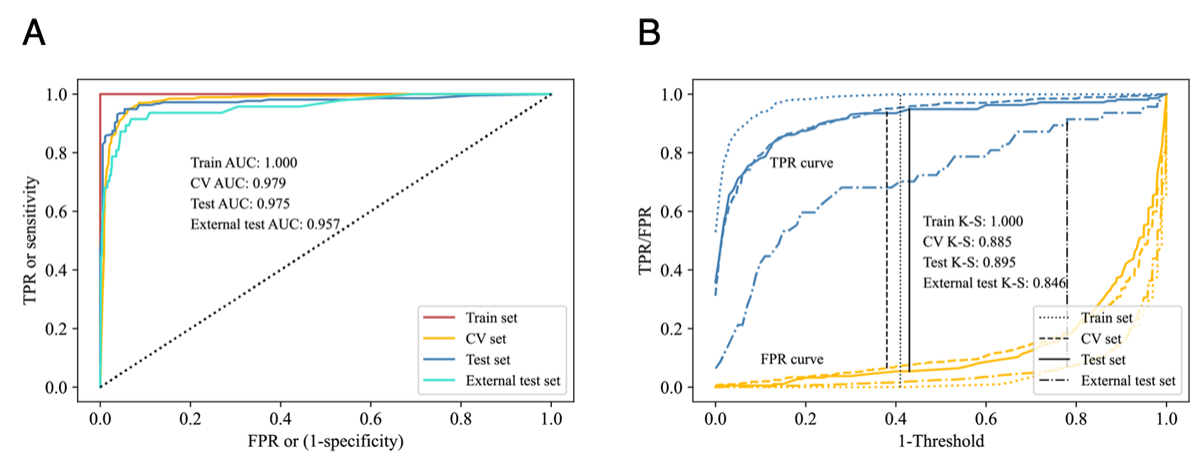
The model performs in differentiating positive and negative samples. (A) Receiver operating characteristic curve of the train, CV, test, and external test sets; (B) Kolmogorov-Smirnov plot of the train, CV, test, and external test sets. AUC: area under the receiver operating characteristic curve; CV: cross-validation; FPR: false positive rate; K-S: Kolmogorov-Smirnov; TPR: true positive rate.

Because MAP, GDP, sexual maturity, BMI, and sex reduced the model’s predictive performance, age, UDVA, and SE of the first 2 years were selected as predictors. T-distributed stochastic neighbor embedding showed that the data points were separable, thus revealing the feasibility for prediction (Figure 7). Compared to logistic regression, the support vector machine, and the k-nearest neighbor algorithm, the random forest algorithm achieved the highest accuracy (Table 2). We adopted a grid search to explore optimal hyperparameters, resulting in 100 estimators with a maximum depth of 19 for each tree. The model achieved an accuracy of 0.939 for the CV set and 0.948 for the test set. The recalls of the CV and test sets were 0.964 and 0.949, respectively, indicating that most positive cases were correctly predicted. According to the AUC score, there was a 97.5% chance for positive samples to have higher probabilities than negative ones in the test set, again demonstrating sufficient model efficacy. The K-S value was 0.895 for the test set, indicating that the most positive and negative samples were successfully differentiated (Figure 8 and Table 3). Our algorithm showed reliable performance in the external validation, with high accuracy (0.971), AUC (0.957), and K-S value (0.846), demonstrating the ability of the model to generalize data.

**Table 2:** Comparison of methods.

Evaluation metrics	Accuracy	5-Fold cross-validation	Precision	Recall	*F*_1_-score
Logistic regression	0.85	0.87	0.86	0.85	0.86
Support vector machine	0.88	0.89	0.92	0.85	0.89
k-Nearest neighbor algorithm	0.88	0.90	0.88	0.89	0.89
Random forest algorithm	0.95	0.96	0.95	0.95	0.95

**Table 3:** Performance of the random forest classifier in the train, CV^a^, and test sets.

Evaluation metrics	Accuracy	Precision	Recall	AUC^b^	K-S^c^
Train set	1.000	1.000	1.000	1.000	1.000
CV set	0.939	0.916	0.964	0.979	0.885
Test set	0.948	0.953	0.949	0.975	0.895

^a^CV: cross-validation.

^b^AUC: the area under the curve.

^c^K-S: Kolmogorov-Smirnov.

## Discussion

### Principal Findings

Real-world research is important for public health and epidemiologic investigations [17]. Our study was based on real-world vision screening records, which included students’ age, sex, location, class, grade, school type, UDVA, NCR, systolic pressure, diastolic pressure, height, weight, and sexual maturity. We best attempted to identify the prevalence pattern of high myopia for children and adolescents in southern China in different years, ages, school types, and cities. We selected age, UDVA, and SE as predictors and generated a random forest algorithm for predicting the onset of high myopia for the first time.

NCR was the main outcome of our research, which corresponds to Chinese large-scale myopia screening practices. Next, we included the combination of UDVA and NCR as a cutoff to screen for high myopia in large-scale school screening, aiming to reduce the gap between screening myopia (based on NCR) and its real-world prevalence. We acknowledge that the gold standard for refraction in children and adolescents is refraction with cycloplegia [14]. Therefore, children suspected with high myopia through our prediction model were warned and referred for a cycloplegic examination. Effective intervention should be conducted immediately to prevent the development of high myopia in the following year.

As confirmed in our study, the prevalence of high myopia in children and adolescents from southern China was higher than the global prevalence (4.0%) [2]. The incidence rate of high myopia rose exponentially with age, with an average growth of >1% annually from the age of 7 to 11 years; increasing prevalence rates of high myopia were observed among individuals in elementary, middle, and high schools. There are several epidemiological studies of myopia in China. Among screened university students in Shanghai (in 2012), 19.5% had high myopia [18]. In Wenzhou City (in 2019), the prevalence of high myopia in elementary, middle, and high school students was 0.95%, 6.90%, and 12.98%, respectively [6]. In Chengdu City (in 2019), the prevalence rate was 1.7% among children aged 3-14 years [19]. These studies were cross-sectional descriptions of the incidence rate of high myopia in different years, cities, and populations, but they did not mention the developmental patterns of high myopia with age. Nevertheless, these studies showed that the prevalence of high myopia had worsened and become a global concern.

We also found a relationship between high myopia and economic development. A published report has shown that economic growth is negatively associated with the vision of school children in China. Every 100% increase in GDP was associated with a 20% increase in the relative risk of moderate to severe visual impairment [20]. Our results were consistent with those of previous studies, with coastal and southern cities (which had higher GDPs) being more affected. This could be because areas with high GDP per capita tend to be wealthier and have more developed school systems. The explanation lies in the link between GDP and education, with intensive education systems as the causal factor [21]. Guangzhou had a relatively low rate of high myopia. We speculate that eye health education has been widely promoted in first-level cities. Parents pay more attention to their children’s vision and have more hospitals available. This suggested that comprehensive myopia prevention and control initiatives are crucial, and the results from Guangzhou are worth promoting in other areas.

We extracted factors such as age, sex, location, MAP, BMI, and sexual maturity from the data set for further analysis and found that age was the most important risk factor, followed by MAP, GDP, sexual maturity, BMI, and sex. Behavioral and environmental factors such as economic development, time spent outdoors, physical activity, green scenery exposure, sunlight exposure, near-work activity, smartphone overuse, diet, and sleep, which have been reported as the main factors of myopia onset and progression [20,22-24] cannot be investigated in large-scale vision screenings. Our analysis of risk factors of high myopia is based on a review by the International Myopia Institute, with the conclusion that the two major risk factors for myopia are high educational pressures and deprivation of time outdoors [25]. We found that age is a significant factor because it implies a higher cumulative exposure to educational pressures. Sexual maturity is not linked with increased age and hence educational exposure. Links to BMI are probably mediated by little time for physical activity and, as a result, little time outdoors. Sex is relevant, but it has a small effect on high myopia. MAP was negatively correlated with high myopia. This is consistent with an ocular blood flow measurement in healthy youths, which showed that patients with high myopia had a lower MAP than those with low myopia and those with emmetropia [26]. We speculated that systemic blood pressure affects ocular blood circulation, leading to high myopia.

To make full use of large-scale data from real-world vision screening and to provide immediate and timely warnings for high myopia onset, we studied the influence of related factors on high myopia incidence based on machine learning. Age was the most significant risk factor. MAP, GDP, sexual maturity, BMI, and sex affected high myopia, but they were not significant predictors and they reduced the predictive performance of the model. Finally, 3 variables—UDVA, SE, and age—were included to develop a random forest algorithm to predict the onset of high myopia in the following year. The results showed that prediction accuracy was >95%, which was reasonable and clinically acceptable. Although Lin’s [9] model could predict SE and the onset of high myopia at 18 years of age as early as 8 years in advance, it was complicated and confined to patients with cycloplegic refraction and annual progression rates. Wang’s prediction model was derived from 2740 cycloplegic autorefraction data [8], showing UDVA, SE, axial length, flat keratometry reading, sex, and parental myopia as predictive risk factors. However, it could not be applied widely because obtaining such a high volume of information through myopia screening is difficult. In this regard, our research was more practical in predicting the onset of high myopia in vision screening because only age, UDVA, and NCR were included.

The strengths of our study include the large sample size and the real-world nature of the data. Owing to some weaknesses of internal validity, study design, incomplete or incorrect data entry, or selection bias [27], many challenges must be addressed when using real-world screening data. Through special sampling and selection of statistical methods, we used real-world large-scale data collected from vision screening to illustrate the prevalence patterns of high myopia in children and adolescents in southern China and efficiently predicted the onset of high myopia in large-scale screening for the first time. We aimed to build a model, which could predict high myopia in children and adolescents, and deduce the relationship between high myopia and different factors to formulate a policy against high myopia. Future research could include a more thorough analysis of the results to incorporate improvements in the methodology.

### Conclusions

High myopia had a high incidence in Guangdong Province. Its onset in children and adolescents was well predicted by the random forest algorithm. Efficient use of real-world data can contribute to the prevention and early diagnosis of high myopia. In the future, we aim to develop a smartphone app that allows people to input their age, UDVA, and NCR to predict the risk of high myopia. The adaptation of network- and smartphone-based myopia prediction platforms will provide a new model for vision screening, and it can dramatically improve vision health in the subsequent generations.

## Abbreviations

AI: artificial intelligence
AUC: area under the receiver operating characteristic curve
CV: cross-validation
GDP: gross domestic product
K-S: Kolmogorov-Smirnov
MAP: mean arterial pressure
NCR: noncycloplegic autorefraction
ROC: the receiver operator characteristic
SE: spherical equivalents
UDVA: uncorrected distance visual acuity
VA: visual acuity

## Data Availability

The data used to support the findings of this study are available from the corresponding author upon request.
